# Preserve and strengthen family to promote mental health

**DOI:** 10.4103/0019-5545.74323

**Published:** 2010

**Authors:** Rajnish Raj

**Affiliations:** Department of Psychiatry, Civil Hospital, Malerkotla, District Sangrur, Punjab, India. E-mail: drrajnish_raj@yahoo.com

Sir,

The Presidential Address of Dr. Ajit Avasthi on the vital role of families in caring and sharing the anxieties, frustrations, and unpleasant emotional experiences of patients suffering from mental disorders is an awakening call to the mental health professionals, non-specialist, paraprofessionals, voluntary organizations, policymakers, and caregivers to have an integrated strategy and holistic approach to decipher the labyrinthine puzzle of mentally sick persons.[1] It is a splendid blend of ancient wisdom and brilliant sparks of modern science. The reflections on the preservation and strengthening of joint family system to promote mental health care are refreshingly illuminating and fascinatingly educative.[2] These reflections help the Indian psychiatrists to remove the gathered gloom of inertia and despair among the psychotics and mentally sick due to utter lack of mental health infrastructure, sharp increase in the cost of treating and rehabilitating mentally sick person’s and alarmingly a sudden decrease in the government expenditure on Health and Family Welfare Programs.[3]

We are living in a society where our physical and mental health defines our existence and any mental disorder in a person amounts to total loss of identity. In such a scenario, family becomes the anchor sheet to prevent his alienation from himself, his work place, colleagues, and peers. The members of the family having great concern and care for such persons alleviate them from their agonizing experiences and restore confidence in them to face the calamity of alienation boldly. They also save them from all kinds of unsavory and sarcastic remarks which arouse hostility and mistrust in them.[4] This caring involvement of the family helps the professionals to treat them in a comfortable and pleasing manner. With a little tact, patience, and dedication, the patients become fully competent and confident to face the agonies in a calm and composed manner.[5]

The Presidential Address, with its breadth of vision and wisdom, will be of immense help to all those who are concerned about the happiness and well-being of patients suffering from mental disorders. It will also enable them to hold their head high when they are able to treat their patients with utmost insight and innovation.
